# Evaluation of major coronary artery Bifurcation angles with digital angiography: A detailed study of prevalence in the Upper Euphrates Basin

**DOI:** 10.12669/pjms.38.3.4782

**Published:** 2022

**Authors:** Gulnihal Deniz, Ahmet Kavakli, Murat Kucukukur, Evren Kose, Ilgin Karaca

**Affiliations:** 1 Dr. Gulnihal Deniz, Assistant Professor, Department of Physiotherapy and Rehabilitation, Faculty of Health Sciences, Erzurum Technical University, Erzurum, Turkey; 2 Prof. Dr. Ahmet Kavakli, MD. Department of Anatomy, Faculty of Medicine, Firat University, Elazig, Turkey; 3 Dr. Murat Kuçukukur, MD. Department of Cardiology, Tepecik Training and Research Hospital, Izmir, Turkey; 4 Prof. Dr. Evren Kose, MD. Department of Anatomy, Faculty of Medicine, Inonu University, Malatya, Turkey; 5 Prof. Dr. Ilgin Karaca, MD. Department of Cardiology, VM Medical Park Bursa Hospital Fevzi Cakmak Street Kırcaali District No: 76, Osmangazi Bursa, Turkey

**Keywords:** Bifurcation, Bifurcation angles, Coronary artery, CX, LMCA, LAD, RCA, PDA, PL

## Abstract

**Objectives:**

To investigate the diversity and average values of bifurcation angles in a large population to help develop new methods.

**Methods:**

One thousand five individuals (504 females, 501 male) who visited the Cardiology Polyclinic of Fırat University Hospital with the complaint of chest pain between 2010 and 2015 were evaluated retrospectively. Bifurcation angle measurements between LMCA-CX, CX-LAD, LMCA-LAD, CX-OM1, CX-OM2, LAD-D1, LAD-D2, RCA-RMD, RCA-RVD and PDA-PL were evaluated in all cases.

**Results:**

Bifurcation angles between LMCA-LAD, LMCA-Cx and LAD-Cx branches with “> 90 wide angle bifurcations”, and Cx-OM1, Cx-OM2, LAD-D1, LAD-D2, RCA-RMD and PDA-PL with “<70 Y type bifurcation angle” were found to be high in male and female individuals. The RCA-RVD in female individuals was “<70 Y-type bifurcation” in 14 (2.8%) people, “> 70-90 T-type bifurcation” in 209 (41.5%) people, and “> 90 wide angle bifurcation” in 281 (55.8%) people. Results for male subjects were compatible with this. The correlations of all angles were examined. Robust positive correlations (p≤0.001) were found for the angular measurements between the main branches and the side branches (Cx-OM1, Cx-OM2, LAD-D1, LAD-D2 and RCA-RMD, PDA-PL).

**Conclusion:**

With the help of developing technology, we believe that all this coronary angiography data will guide bifurcation stent techniques, which are essential alternatives to bypass.

## INTRODUCTION

Coronary artery stenosis and associated clinical events [acute coronary syndrome (unstable angina pectoris, ST elevation myocardial infarction, non-ST elevation myacardial infarction), sudden death] is the most important cause of mortality worldwide. In its treatment; percutaneous stent placement in eligible patients has become increasingly common in daily use.^1^ Compared to the bypass; the mortality rate of percutaneous coronary intervention is relatively low, practical and fast, causing it to be increasingly preferred in coronary artery disease in recent years. Bifurcation lesions are an important problem in the treatment of coronary artery disease by the percutaneous route. Approximately 15-20% of percutaneous interventions performed all over the world are bifurcation lesions. Which is the lesion of the main coronary artery, containing an important side branch, occurring at the origin of the side branch or adjacent to it.^2^ Bifurcation angle is an important factor in determining the choice of interventional technique, predicting the success of the procedure and follow-up results. But in the literature; there are not enough studies about the frequency and mean values of coronary artery bifurcation angles. Therefore, the ideal percutaneous treatment approach has not been determined.^3^

Our aim, in order to ensure the success of the procedure and patient follow-up problems in stent applications, it is determining the diversity and mean values of bifurcation angles and which is being helped to develop appropriate stent designs and new methods. The most important point in the treatment of bifurcation lesions by percutaneous coronary intervention is necessary to determine an apart and most appropriate strategy for each lesion. In the literature, many special techniques are being developed for bifurcation lesions such as Culotte, Crush, Tap stenting techniques. In addition, studies on this subject are still not clear.^4^,^5^ With this study, we aimed to contribute to the literature by evaluating the diversity, averages and correlations of bifurcation angles.

## METHODS

A total of 1005 patients who complained of chest pain and underwent diagnostic coronary angiography between 2010 and 2015 at the Cardiology Polyclinic of Firat University Hospital were evaluated retrospectively. Of the 1005 cases in our study 504 female and 501 male patients were recruited. Cardiomyopathy, heart failure, left ventricular hypertrophy, dilatation, atrial fibrillation, valvular or congenital heart disease, active connective tissue disease, symptomatic arrhythmia, and branch block, patients with chronic liver and chronic renal failure and by-pass patients were excluded from the study. Coronary angiography procedure included Philips Integris Allura 9 C monoplane diagnostic cardio-vascular, interventional procedures poly diagnost G. Stand digital imaging (2006, The Nederland B.V) Left heart catheterization with the Judkins technique, right and left coronary angiographies were performed selectively. Coronary angiography images were taken in LAO caudal (left anterior oblique caudal), RAO caudal (right anterior oblique caudal), LAO cranial (left anterior oblique cranial) and RAO cranial (right anterior oblique cranial) positions. All measured parameters were opened in the “ExtremePACS cardiac viewer” program and measured with a digital goniometer in the same program.

### Measured Parameters:

**Fig.1:**
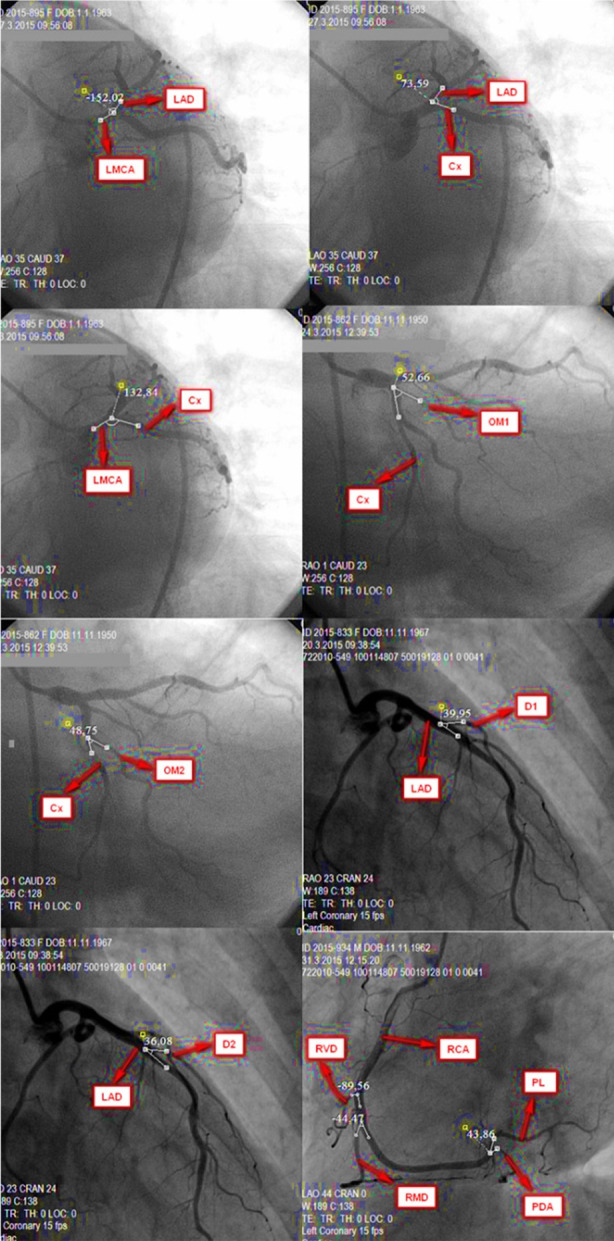
Bifurcation angle measurements of LMCA, LAD, Cx, OM1, OM2, D1, D2, RCA, RMD, RVD, PDA, PL

1. The bifurcations of LMCA, LAD and Cx were examined in 30-60º LAO and 10-45º caudal angiographies. Angle between branches LMCA-LAD, LAD-Cx, LMCA-Cx were measured.
2. The bifurcations of the Cx and its branches were examined in the angiographies taken at 10-45º RAO and 10-30º caudal position. Angle between branches Cx-OM1, Cx-OM2 were measured.
3. The bifurcations of the LAD and its branches were examined in the angiographies taken at the 5-30º RAO and the 20-45º cranial position. Angle between branches LAD-D1, LAD-D2 were measured.
4. The bifurcations of the RCA and its branches and the branches of the RVD with RCA were examined in the angiographies taken at 30-45º LAO and 15-25º cranial position. Angle between branches RCA-RMD, PDA-PL were measured (Fig.1).

SPSS 22.0 package program was used for the statistical analysis of the study. Data obtained in the study were given as mean±standard deviation (X±SD). Independent Student-t test was used to compare parameters showing normal distribution and homogeneity for quantitative data. Crosstabs table was applied to classify the data and analyze the relationships between them. Pearson Correlation test was used to examine the relationships between parameters. Statistical values of p≤0.05 were considered significant.

### Ethical Approval

This study was conducted after approval from the Non-Interventional Research Ethics Committee of Firat University (Decision No.: 83814, dated March 18, 2015).

### Patients’ consent

As the study was designed retrospectively, data was collected from clinical archives after ethical approval.

## RESULTS

There were 504 female (min-max 46-73) and 501 male (min-max 44-72) participants in our study. Mean age values of the individuals 60.06±0.34 years and 59.28±0.34 years in the female and male group, respectively (p=0.108, p>0.05). In this study, bifurcation angles between LMCA-LAD, LMCA-Cx, LAD-Cx, Cx-OM1, LAD-D1, RCA-RVD and PDA-PL were measured in 504 women and 501 men. While measuring the bifurcation angles between Cx-OM2, LAD-D2 in 502 women and 499 men (there were no branches OM2 and D2 in two females and two males). Whereas measuring bifurcation angles between RCA-RMD in 416 women and 421 men; The bifurcation angles between RCA-RMD could not be measured in 88 female and 80 male individuals inasmuch as there was no RMD branch.

Bifurcation angles between Cx-OM1 and Cx-OM2 branches were found to be statistically more significant and higher in women than in men (p≤0.01). Bifurcation angles between LMCA-LAD, LAD-Cx, PDA-PL branches were found to be statistically significant and higher in men than in women (p≤0.05), (Table-I). Coronary artery bifurcation angles measured in our study were classified into three main groups.

1. <70 Y-type bifurcation angles,
2. >70-90 T-type bifurcation angles,
3. >90 Wide-angle bifurcation angles.^6^, ^7^

**Table-I:** Comparison of Measured Bifurcation Angle Measurements in All Cases of Female and Male Cases.

Bifurcation Angle Measurements	Gender	n	Min-Max	X±SD	p
LMCA-LAD	Female	504	76.50-190.20	156.35±0.65	0.013*
	Male	501	81.29-186.50	153.90±0.72	
LMCA-Cx	Female	504	66.80-161.80	106.34±0.57	0.875
	Male	501	43.48-154.55	106.21±0.63	
LAD-Cx	Female	504	32.30-165.40	96.85±0.62	0.019*
	Male	501	42.60-160.10	99.04±0.68	
Cx-OM1	Female	504	24.28-102.96	50.52±0.52	0.008**
	Male	501	28.50-100.48	48.60±0.49	
Cx-OM2	Female	502	21.50-108.31	48.69±0.47	0.002**
	Male	499	23.63-107.59	46.68±0.45	
LAD-D1	Female	504	21-80.94	43.32±0.42	0.105
	Male	501	22.60-95.40	42.38±0.42	
LAD-D2	Female	502	23.70-103.10	45.51±0.46	0.331
	Male	499	16.80-89.69	44.89±0.43	
RCA-RVD	Female	504	42.30-128.66	90.16±0.42	0.742
	Male	501	32.70-133.18	90.37±0.48	
RCA-RMD	Female	416	18.20-110.20	46,60±0,44	0.439
	Male	421	22.47-93.20	47.09±0.43	
PDA-PL	Female	504	23.54-89.72	46.48±0.29	0.015*
	Male	501	26.76-108.50	47.68±0.39	

X ± SD: Values are given as mean ± standard deviation.

* p ≤0.05,

** p ≤0.01

LMCA: a. coronaria sinistra, LAD: r. interventricularis anterior, Cx: r. circumflexus, OM1: r. marginalis sinister, OM2: r. posterior ventriculi sinistri, D1-D2: r. lateralis, RCA: a. coronaria dextra, RVD: r. ventriculus dexter, RMD: r. marginalis dexter, PL: r. posterolateralis dexter.

The bifurcation angles between the LMCA-LAD, LMCA-Cx and LAD-Cx branches were found to be a high rate of “>90 wide angle bifurcation” in female and male individuals. The bifurcation angles between the branches of Cx-OM1, Cx-OM2, LAD-D1, LAD-D2, RCA-RMD and PDA-PL were found to be a high rate of “<70 Y-type bifurcation angle” in female and male individuals. The bifurcation angle between RCA-RVD branches is in female individuals; while it was found as “<70 Y-type bifurcation” in 14 (2.8%) people, “>70-90 T type bifurcation” in 209 (41.5%) people and “> 90 wide angle bifurcation” in 281 (55.8%) people, similarly found in male subjects.

The correlations of bifurcation angle measurements with each other were examined. Negative correlations were found in bifurcation angles between LMCA-LAD, LMCA-Cx and LAD-Cx branches in females. Positive correlations were found between the bifurcation angles between Cx-OM1, Cx-OM2, LAD-D1 and LAD-D2 branches in females (p≤0.001), (Table-II).

**Table-II:** Correlation Analysis of Bifurcation Angle Measurements between A. Coronaria Sinistra and Its Branches.

		LMCA-LAD		LMCA-Cx		LAD-Cx		Cx-OM1		Cx-OM2		LAD-D1		LAD-D2	
		r	p	r	p	r	p	r	p	r	p	r	p	r	p
Female	LMCA-LAD			-0.402	0.00**	-0.518	0.00**	-0.073	0.103	-0.019	0.675	0.031	0.483	-0.025	0.580
	LMCA-Cx	-0.402	0.00**			-0.363	0.00**	-0.013	0.770	0.015	0.733	0.124	0.005**	0.098	0.029*
	LAD-Cx	-0.518	0.00**	-0.363	0.00**			0.034	0.450	0.035	0.429	-0.059	0.186	-0.010	0.817
	Cx-OM1	-0.073	0.103	-0.013	0.770	0.034	0.450			0.480	0.00**	0.205	0.00**	0.172	0.00**
	Cx-OM2	-0.019	0.675	0.015	0.733	0.035	0.429	0.480	0.00**			0.197	0.00**	0.192	0.00**
	LAD-D1	0.031	0.483	0.124	0.005**	-0.059	0.186	0.205	0.00**	0.197	0.00**			0.553	0.00**
	LAD-D2	-0.025	0.580	0.098	0.029*	-0.010	0.817	0.172	0.00**	0.192	0.00**	0.553	0.00**	
Male	LMCA-LAD			-0.448	0.00**	-0.523	0.00**	-0.131	0.003**	-0.028	0.538	-0.003	0.938	-0.074	0.100
	LMCA-Cx	-0.448	0.00**			-0.368	0.00**	0.051	0.253	0.063	0162	-0.056	0.212	0.076	0.089
	LAD-Cx	-0.523	0.00**	-0.368	0.00**			0.076	0.088	0.018	0.695	0.069	0.121	-0.007	0.883
	Cx-OM1	-0.131	0.003**	0.051	0.253	0.076	0.088			0.500	0.00**	0.048	0.287	0.122	0.007**
	Cx-OM2	-0.028	0.538	0.063	0162	0.018	0.695	0.500	0.00**			0.074	0.097	0.017	0.705
	LAD-D1	-0.003	0.938	-0.056	0.212	0.069	0.121	0.048	0.287	0.074	0.097			0.527	0.00**
	LAD-D2	-0.074	0.100	0.076	0.089	-0.007	0.883	0.122	0.007**	0.017	0.705	0.527	0.00**	
Total	LMCA-LAD			-0.426	0.00**	-0.524	0.00**	-0.095	0.003**	-0.016	0.620	0.018	0.572	-0.047	0.139
	LMCA-Cx	-0.426	0.00**			-0.365	0.00**	0.020	0.531	0.040	0.207	0.035	0.274	0.087	0.006**
	LAD-Cx	-0.524	0.00*	-0.365	0.00**			0.049	0.124	0.019	0.546	0.001	0.971	-0.011	0.735
	Cx-OM1	-0.095	0.003**	0.020	0.531	0.049	0.124			0.494	0.00**	0.136	0.00**	0.150	0.00**
	Cx-OM2	-0.016	0.620	0.040	0.207	0.019	0.546	0.494	0.00**			0.144	0.00**	0.112	0.00**
	LAD-D1	0.018	0.572	0.035	0.274	0.001	0.971	0.136	0.00**	0.144	0.00**			0.542	0.00**
	LAD-D2	-0.047	0.139	0.087	0.006**	-0.011	0.735	0.150	0.00**	0.112	0.00**	0.542	0.00**	

LMCA: a. coronaria sinistra, LAD: r. interventricularis anterior, Cx: r. circumflexus, OM1: r. marginalis sinister, OM2: r. posterior ventriculi sinistri, D1-D2: r. lateralis.

* p≤0.05,

** p≤0.01

In male individuals; negative correlations were found between the bifurcation angles between the LMCA-LAD, LMCA-Cx and LAD-Cx branches. A negative correlation was found in bifurcation angles between LMCA-LAD and Cx-OM1 branches in male subjects. Positive correlations were found in bifurcation angles between Cx-OM1 and Cx-OM2 and LAD-D1 and LAD-D2 branches in male subjects. When the correlation of bifurcation angles in all cases was examined, it was seen that it was compatible with the correlation scores in male and female subjects. A negative correlation was obtained between the bifurcation angles between the LMCA-LAD branches and the bifurcation angles between the LMCA-Cx, LAD-Cx and Cx-OM1 branches (Table-II).

There were positive correlations between bifurcation angle measurements between Cx-OM1 with Cx-OM2, LAD-D1 with LAD-D2 among themselves. In addition, positive correlations were found in bifurcation angles between the branches of Cx-OM1 with Cx-OM2, LAD-D1 and LAD-D2 branches (Table-II).

No correlation was found between bifurcation angle measurements between RCA-RVD branches and bifurcation angle measurements between RCA-RMD and PDA-PL branches in females (p>0.05). However, a positive correlation (p≤0.001) was observed between the bifurcation angle measurements between the RCA-RMD branch and the bifurcation angle measurements between the PDA-PL branch. Correlations between bifurcation angle measurements of RCA-RVD, RCA-RMD and PDA-PL branches were not significant in male. Considering the correlation in the mean values of bifurcation angles of all cases, it was seen that there was a positive correlation (p≤0.001) between bifurcation angle measurements between RCA-RMD branch and bifurcation angle measurements between PDA-PL branch.

## DISCUSSION

As a result of the developing world and the changes it has brought to social life, atherosclerotic coronary artery disease has become one of the main causes of mortality and morbidity. Bifurcation distributions in coronary arteries are highly prone to the development of atherosclerotic disease. Bifurcation lesions in coronary arteries are generally defined as coronary artery disease that includes the “important side branch” ostium or its neighborhood. Coronary artery bifurcation lesions is an important problem in cardiology practice and the issue of how the ideal percutaneous treatment approach would be in such cases is still being debated. New methods are applied every period and case-specific techniques are developed.^8^

While bifurcation lesions increase the duration of the procedure and the possibility of complications, especially in patients who are planned percutaneous coronary intervention, and reduce the success rate of percutaneous coronary intervention. In the guidelines published by world-renowned institutions related to interventional cardiology, it has been repeatedly stated that the coronary artery lesion to be intervened includes bifurcation, which increases the risk of the procedure. Furthermore, many studies have shown that restenosis and stent thrombosis are observed more frequently in the follow-up of patients who underwent percutaneous coronary intervention for bifurcation lesions. As a result, negative results may be inevitable in patients with bifurcation lesions.^9^

In recent years, the fact that approximately 15-20% of percutaneous coronary interventions are applied to coronary artery bifurcation lesions may help us understand the extent of the problem. Recent studies and new treatment techniques also show how often bifurcation lesions are encountered and better results are obtained in clinical follow-up.^10^

New techniques are planned for the percutaneous treatment of coronary artery bifurcation lesions in interventional cardiology each day. Besides, cases with percutaneous treatment indication are among the lesions that have specific difficulties. Also, evaluation of bifurcation angle before percutaneous coronary intervention; It has been observed that it contributes greatly to the success of the process, as it can clearly show the composition and borders of the plate that causes stenosis.^10^-^12^

Bifurcation angle is an absolute factor in determining the interventional technique to be chosen, in predicting the success of the procedure and follow-up results. In interventions applied to bifurcation lesions, separate percutaneous treatment should be applied to the main and side branches. In interventions applied to bifurcation lesions percutaneous treatment, should be applied to the main and side branches separately. But in the literature; there are not enough studies about the frequency and mean values of coronary artery bifurcation angles. Therefore, the ideal percutaneous treatment approach has not been established.^13^-^15^

 Studies on bifurcation lesions and treatment methods in the literature shows the average age of the cases is between 55-65. It was observed that the number of male cases was higher in studies.^16^-^20^ In our study, the number of men and women were studied in a balanced way (504 female 60.06±0.34 years, 501 male 59.28±0.34 years and p=0.108). A large population was evaluated and the diversity of bifurcation angles and their mean values were determined. These values were first compared in male and female individuals. Most of the studies in the literature didn’t compare gender and studies were mostly conducted on male individuals.^18^-^20^ Temov and Sun^21^ found that the bifurcation angle between the LAD-Cx was significantly higher in men than in women, similar to ours (p=0,003). In our study; many angles which compared in male and female individuals differed statistically (Table-I). It is thought that the gender factor should not be ignored in all studies on bifurcation lesions and their treatment in the literature.

Kawasaki et al.^9^ evaluated the coronary artery bifurcation angles with multi-slice computed tomography in 209 patients and became one of the firsts in Japan in this regard. Craiem et al.^22^ evaluated the coronary artery bifurcation angles with multi-slice computed tomography and found that there were wider angles in the atherosclerotic group. Sun^23^ found higher bifurcation angle in male patients than in normal individuals. Kurt et al.^24^ evaluated bifurcation angle measurements between LAD and Cx branches and 77% of them had bifurcation angles below 70°. Sun et al.^25^ reported that 89% of LAD-Cx were in the wide-angle bifurcation class. Sun^23^ reported that 91% of LAD-Cx were greater than 80º. In our study, the group of accumulation was determined by grouping the bifurcation angles according to the special bifurcation stents used in the treatment. When we look at the bifurcation between main branches of LMCA; It was determined that the angles in male and female cases were higher than 90º, that is, in the wide-angle bifurcation class. In the literature, the bifurcation angles between the main branches of LMCA have also been found as obtuse angles.^9^ In our study; When the bifurcation angle measurements between LAD-D1, LAD-D2, Cx-OM1, Cx-OM2, RCA-RMD and PDA-PL branches are grouped; It has been stated that the bifurcation angles in male and female individuals are less than 70º, that is, they are in the Y-type bifurcation angles class. When we examined the studies in the literature on bifurcation angle measurements between branches LAD-D1, LAD-D2, Cx-OM1, Cx-OM2 and PDA-PL, it was found to be similar to ours.^24^ In addition, bifurcation angle measurements between RCA-RMD and RCA-RVD branches were not found in literature reviews. When the bifurcation angle measurements between RCA-RVD branches are grouped; It was observed that the bifurcation angles in male and female individuals were mostly grouped in both the T-type bifurcation class and the wide angle bifurcation angle class. As stated in the literature, such classifications will be very effective in choosing the appropriate techniques for the diagnosis and treatment of bifurcation lesions.^7^

In our study, the correlations of bifurcation angle measurements with each other were also examined. When bifurcation angles between LMCA-LAD, LMCA-Cx and LAD-Cx branches are evaluated in female and male individuals, one of them increased while the other decreased; that is, they had negative correlations (p≤0.001). A very important finding is that the bifurcation measurements between the main branch and side branches (Cx-OM1, Cx-OM2, LAD-D1 and LAD-D2) had positive correlations (p≤0.001). According to these correlations, it was concluded that the narrowness or width of an angle of the side branch also affects the angle of the other side branch. When the correlations between RCA and its branches were examined, there was a positive correlation (p≤0.001) between the bifurcation angle measurements between the RCA-RMD branch and the bifurcation angle measurements between the PDA-PL branch. When the correlations between RCA and its branches were examined, there was a positive correlation (p≤0.001) between the bifurcation angle measurements between the RCA-RMD branch and the bifurcation angle measurements between the PDA-PL branch. However, sufficient data on this subject could not be found in the literature.

### Limitations of the study

First, the number of samples and duration should have been increased to achieve more accurate results. Second, we only focused on the diversity and mean values of bifurcation angles in this study, atherosclerotic lesions should be grouped and included in the next study. Thus, it is thought that it will help in determining and interpreting the invasive intervention technique by comparing normal and lesioned bifurcation angles.

## CONCLUSION

It was determined that gender factor is important in coronary artery bifurcation angles and, female individuals should be evaluated, along with male individuals. The bifurcation angles between the main branches of the LMCA and the first branch of the RCA are greater than 90º (wide angle bifurcation), the bifurcation angles between the side branches are less than 70º (Y-type bifurcation), that is, it has been observed that the main branches are wide-angle bifurcation and the side branches are narrow angle bifurcation. Considering that wide-angle bifurcation lesions in the clinic are a bigger problem than narrow-angle bifurcation lesions, the importance of special stenting techniques in bifurcation lesions of the main branches have been proven with our study. As an important correlation was concluded that the narrowing and width of the bifurcation angles of the lateral branches positively affected the next branch. Due to the limited number of studies on RCA, we believe that bifurcation angle measurements of all RCA branches measured in detail in our study will contribute to the literature. With the help of developing technology, we believe that all this coronary angiography data will guide bifurcation stent techniques, which are essential alternatives to bypass.

### Note

This study is the completed PhD thesis of Gulnihal Deniz.

### Authors’ Contribution:

**GD:** Planned the study, collected and analyzed the data, manuscript writing.

**AK:** Interpreted the results, and prepared the draft.

**MK:** Did statistical analysis & manuscript writing.

**EK:** Helped to review and editing the manuscript.

**IK:** Did review and final approval of manuscript.

All of the authors take the responsibility and is accountable for all aspects of the work to ensure the accuracy and integrity of the work are appropriately investigated and resolve
